# Anti-bias training for (sc)RNA-seq: experimental and computational approaches to improve precision

**DOI:** 10.1093/bib/bbab148

**Published:** 2021-05-06

**Authors:** Philip Davies, Matt Jones, Juntai Liu, Daniel Hebenstreit

**Affiliations:** Daniel Hebenstreit’s Research Group University of Warwick, CV4 7AL Coventry, UK; Daniel Hebenstreit’s Research Group University of Warwick, CV4 7AL Coventry, UK; Physics Department, University of Warwick, CV4 7AL Coventry, UK; University of Warwick, CV4 7AL Coventry, UK

**Keywords:** RNA-seq, bias, software, modeling, gene expression, quantitation

## Abstract

RNA-seq, including single cell RNA-seq (scRNA-seq), is plagued by insufficient sensitivity and lack of precision. As a result, the full potential of (sc)RNA-seq is limited. Major factors in this respect are the presence of global bias in most datasets, which affects detection and quantitation of RNA in a length-dependent fashion. In particular, scRNA-seq is affected by technical noise and a high rate of dropouts, where the vast majority of original transcripts is not converted into sequencing reads. We discuss these biases origins and implications, bioinformatics approaches to correct for them, and how biases can be exploited to infer characteristics of the sample preparation process, which in turn can be used to improve library preparation.

## Introduction

RNA-seq has become one of the most important tools in molecular biology. It allows straightforward measurement of RNA expression levels in transcriptome-wide fashion. It is now available in countless variants that allow sequencing of different types of RNAs, from different starting materials, using different experimental approaches, and more [1]. Although developed early [2], RNA-seq from single cells (scRNA-seq) increased dramatically in its popularity recently [3]. The power of scRNA-seq lies in its ability to potentially visualize variability that is masked by the ensemble averaging of standard RNA-seq; it can be used to identify allelic exclusion based on single-nucleotide polymorphisms [4] and can reveal non-genetic heterogeneity. The latter is believed to be important in diseases [5] and can offer insights into transcriptional mechanisms [6, 7].

In this review, we will discuss current limitations of RNA-seq with respect to its main application of quantifying transcript abundances. Since this is particularly relevant for absolute quantitation, we will explore how technical noise and biases reduce both sensitivity and precision of (sc)RNA-seq specifically, and will discuss novel insights in this regard.

## From RNA to sequencing reads

The main goal of RNA-seq in most contexts is the accurate quantification of the original RNAs’ abundances in a sample, whether that refers to ‘bulk’ RNA from a homogenized cell population, or single cells. In practice, this amounts to correctly interpreting the number of sequencing reads that are obtained for each transcript. This problem is non-trivial due to several confounding factors preventing precise quantification, most of which are owed to the complexity of RNA-seq sample preparation.

Several steps are necessary to convert the RNAs in cell lysates into sequencing reads. Common to the vast majority of protocols are selecting which RNA is to be sequenced, the cDNA production steps of reverse transcription (RT) (often referred to as first-strand synthesis) and second-strand synthesis. The reason for selecting RNA to be sequenced is that the vast majority of RNA in cell lysates is ribosomal RNA, which is normally undesired. Removing it allows for more reads to be used towards the detection of less abundant RNA species of interest, such as mRNA. This is achieved by removing rRNA (‘ribodepletion’) or positive selection of RNAs of interest. RNA is replaced with DNA because RNA is problematic to work with; it is subject to degradation through RNases and metal ion catalyzed hydrolysis at higher temperatures. It has a propensity to form secondary structures and cannot easily be amplified due to a lack of suitable enzymes and its compromised stability during thermal cycling. Synthesis of the second cDNA strand is necessary to enable adapter ligation for next generation sequencing, unless special adaptations are used [8]. Other protocols use the RT step to add adapter sequences directly, for instance by using a RT primer with overhanging adapter sequences. This idea is taken further in scRNA-seq protocols where the RT primer is often oligo-(dT)s (to capture polyadenylated mRNAs) with an overhang including adapter sequences, cell barcodes and unique molecular identifiers (see section UMIs e.g. 10x Chromium [9], Drop-seq [10] or InDrop [11]).

The only RNA-seq strategy that avoids cDNA conversion is direct RNA sequencing, as implemented by the ill-fated Helicos sequencing machine [12] or nanopore sequencing [13]. The latter is promising as a future technology producing long reads for single molecules; it records the base sequence of individual nucleic acid strands as they are electrophoretically pulled through channels in a membrane. The system is plagued with high error rates, though, and most studies have exploratory character and/or use additional second generation (e.g. Illumina) sequencing to bolster sequencing quality [14].

RNA-seq libraries are usually fragmented by various means and size-selected in order to produce more sequencing reads at optimal length. This can occur before or after cDNA production. Direct fragmentation of RNA often uses metal-ion catalyzed hydrolysis at high temperatures (e.g. TruSeq) and cDNA fragmentation often uses physical methods (e.g. sonication) or enzymatic methods. ‘Tagmentation’ is a convenient enzymatic way to combine fragmentation and adapter ligation [15]. It uses transposase Tn5 to internally cleave double-stranded DNA and ligate oligonucleotides to both resulting ends in the same reaction. The material is usually further amplified by PCR. Often, an extended first PCR cycle is used to synthesize the second-strand. An alternative to PCR is linear amplification by *in vitro* transcription (IVT), as implemented by the CEL-seq protocol [16].

**Figure 1:**
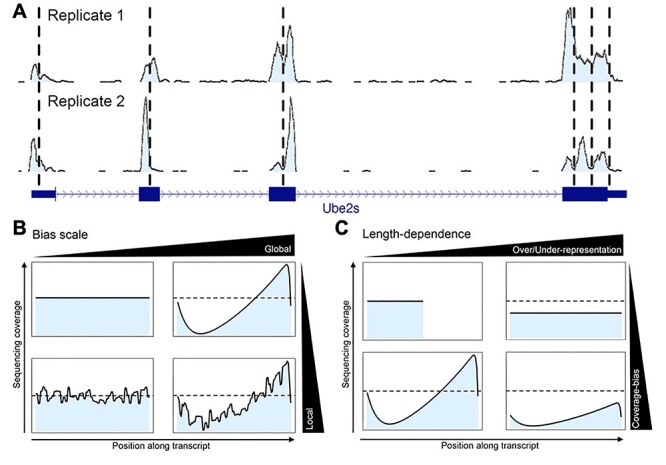
(**A**) RNA-seq coverages by sequencing read along an example gene (Ube2s) for two biological replicates. Abrupt changes in exonic read densities (vertical dashed lines) often coincide across samples, suggesting that the local sequence environment is responsible for this type of bias. Data from GEO, accession numbers GSM710183 and GSM710184. (**B**) RNA-seq coverage along a typical transcript can be subject to bias at different scales; the schematic illustration depicts an absence of visible bias (top left), a local bias (bottom left), a global bias (top right) and a combination of the latter two (bottom right). (**C**) Global bias depends on transcript lengths. Schematic illustration of the length-dependent effects compared to a short reference transcript with no visible bias (top left). Upon considering longer transcripts in the same sample, a global bias can appear (bottom left), which does not necessarily lead to a skewed overall representation of the transcripts (the dashed horizontal line indicates average coverage equal to the reference). However, different lengths often do lead to unequal representation of transcripts due to global bias that might be invisible or visible in terms of coverage (top and bottom right, respectively).

Each of these steps can skew the representation of original transcripts by sequencing reads. It is worth noting that there is a difference between variability and bias. Statistically, the *average* of a repeatedly sampled value needs to deviate from the true value to make it an actual bias; random variation *per se* is not enough. Biases in RNA-seq can have very different effects and it is important to understand, classify and quantify these. Two key properties that help categorize biases are their *scale* (local – bias is specific to one gene or individual positions, or global – bias occurs across genes in a systematic overall pattern) and their *visibility* (can be seen on a coverage plot, e.g. Figure 1A), which are explained in more detail below. These properties are not always independent.

In the next section, we introduce the two major methods for quantifying the abundance of RNA in a sample. We discuss how the sample preparation process introduces bias for coverage-based approaches, avoids these biases for UMI-based approaches, and how these approaches compare otherwise.

## Quantitation approaches

Read numbers alone are not sufficient to quantify the abundance of RNA in a sample and need to be expressed in terms of transcript numbers to draw conclusions about biological processes in many cases. Here, we discuss the two main approaches, read-coverage and UMIs, and their strengths and limitations.

### Coverage-based approaches

Coverage (the number of sequencing reads that align to known reference bases)-based approaches have characteristic biases which are likely to affect quantitation of expression levels. These can occur on a well-studied *local* scale, or an as yet under-characterized *global* scale.

It is generally assumed that expected sequencing read numbers for a particular transcript are proportional to its length, i.e. a linear relationship, giving rise to the RPKM/FPKM (reads/fragments per kilobase transcript length per million total sequencing reads) or transcripts per million (TPM) measures [17]. These have been recognized to be inadequate in their original conception and are frequently subjected to various bias correction algorithms, although the fundamental notion of length-proportionality is usually kept [18]. It is worth noting that application of correction algorithms subverts the physical unit/dimensions character of their names.

#### Local biases

If the sequencing read density is plotted along gene bodies, usually a spikey peak landscape emerges (Figure 1A). Frequently, abrupt changes in coverage coincide for independent replicate samples, suggesting that the local sequence environment causes an actual bias and not just experimental variability (Figure 1A). This corresponds to *local* bias that is highly *visible* (Figure 1B, bottom left). Its causes are debated, but are likely to include RNA secondary structure, non-uniform hydrolysis of RNA, RNA binding proteins and others [19, 20]; most of these factors are speculated to prevent cDNA production at certain spots and/or stop cDNA production in the spots’ vicinity, thus causing free ends that might facilitate adapter ligation (unless tagmentation is employed).

A potentially powerful experimental solution to this problem could be provided by reverse transcriptases found in mobile group II introns. These introns are retroelements that are mainly found in prokaryotes, fungal and plant organellar genomes. They consist of an autocatalytic intron RNA and an intron-encoded reverse transcriptase which act jointly to excise the intron and reverse-splice it into DNA, thereby propagating themselves. Engineered versions of such reverse transcriptases have been shown to have high fidelity and processivities, and are thermostable, which permits increased incubation temperatures during RT, thereby reducing RNA secondary structures. In addition, they exhibit template switching activity that foregoes the need to ligate primers or adapters to the RNA [21]. However, increasing temperatures decreases the stability of RNA [22] and reduces processivity [23]. Therefore, a delicate balance has to be struck between minimizing secondary structures and degradation while maximizing processivity.

The picture is not entirely conclusive, though; RNA-seq libraries that are based on poly-A tail priming still feature many (often short) genes with peak-valley-peak formations where the 5′ peaks are larger than the 3′ ones ( [23] and Figure 4A). This appears hard to reconcile with the idea of obstacles to RT.

In general, this local type of bias does not necessarily have a strong effect on quantification [24]. Although the estimation of splice variant abundances can be skewed depending on differential inclusion of individual peaks or valleys, the local variability might average out for longer RNAs. A related local bias concerns the apparent non-uniform binding of random oligonucleotides [25], which are used in some protocols to prime RT. This bias manifests as unequal nucleotide frequencies at the ends of sequencing reads, which probably affects coverage similarly as the aforementioned examples for local biases.

#### Global biases

A reasonably well understood and intuitive bias arises from reduced fragmentation efficiency close to the ends of DNA fragments. Tagmentation requires a minimum sequence of ~10 bases on either end of the integration sites [15]. Similarly, physical fragmentation methods, such as sonication, probably exert higher tensile stress on longer strands which facilitates breakage in longer DNA. The results are fewer sequencing reads from regions with ineffective fragmentation, which causes noticeable dips in coverage at the ends of transcripts. However, fragmentation bias is more complex than it seems at first glance; cDNA production might stop before the end of the transcript is reached (see below), which potentially biases fragmentation internally, making the bias less visible and harder to correct. In addition, even in the absence of internal fragmentation bias, RNAs that are too short for effective fragmentation will become depleted. Thus, a *global* bias is introduced that affects transcript representation in a non-linear, length-dependent way. This is an example of a bias that is visible and has both local and global effects. In general, different combinations of local and global bias might occur (Figure 1B, bottom right). Potentially, a global bias severely skews the representation of transcripts, e.g. by underestimating long ones [20], but is *invisible* as far as coverage profiles are concerned (Figure 1C).

An important example for global bias is the unequal amplification of different sequences by PCR at exponential rates [26]. This has been of particular concern for scRNA-seq, due to the many PCR cycles that are required. This bias is well recognized and efforts have been made to tackle it by using IVT [16] and by employing UMIs as described below [27], albeit these measures are not compatible with all RNA-seq protocols. In fact, the protocols themselves introduce strong global biases which warrant a closer examination.

#### The origins of global bias

**Figure 2:**
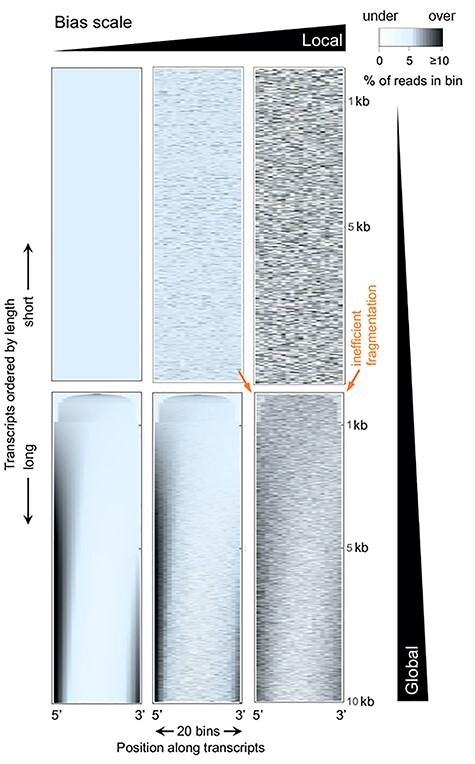
Heatmap representation of bias. Data were simulated to contain no bias (top left) or increasing levels of local bias (left to right) and/or global bias (bottom row). Each heatmap displays transcripts spanning 100 bases to 10 kb that are aligned at 5′ and 3′ ends and are ordered from shortest to longest (top to bottom, respectively). Read coverages are indicated by color in 20 bins along transcripts (color key, top right). The global bias exhibits non-linear length-dependent scaling, from uniform coverage, to 5′ bias, to a bimodal distribution (dark streaks). Typical underrepresentation of transcript ends due to inefficient fragmentation is indicated by orange arrows (shown for one of the three bottom plots affected by it).

Heatmaps are a convenient way to simultaneously depict the local and global scale of a visible bias [23]; if the density of sequencing reads along RNAs is color-coded and RNAs are ordered by length, patterns emerge. A selection of simulated datasets illustrates this for varying degrees and types of bias (Figure 2). The local bias component appears as the noisy color fluctuations throughout the center and right images (Figure 2) and which resemble video noise. Fragmentation dips at either RNA ends, which have both local and global character as explained above, are visible for the bottom row of images (Figure 2). Finally, a strong length-dependent (non-linear) global bias is present in this example as black vertical streaks at the ends of long transcripts (Figure 2). This type of bias appears in similar fashion in many actual datasets and is due to the RNA-seq library preparation process; it has substantial effects on quantification – what is its origin?

One difference in the sample preparation process is how RNA is selected to be sequenced; this can cause bias through the mechanism of RNA degradation. As mentioned earlier, RNA (compared to DNA) is an inherently unstable molecule, with a reactive 2′ hydroxyl group which (when deprotonated) can attack the neighboring phosphodiester bond [22], resulting in self cleavage. This degradation is complicated further by the presence of RNase’s in both the surrounding environment and endogenously in the sample being studied [22]. The instability of mRNA is one of the reasons why mRNA is converted into DNA in the early stages of most protocols – however some degradation is likely to still occur in the process.

A visible *global* bias can be introduced with RNA degradation when RNA is selected from one end of the transcript (poly-(A) + selection) [28]. If one mRNA strand is split in two, and only one strand is selected for (the poly-(A) + strand), then the other will be missed. If the assumption is made that cuts in mRNA occur with equal probability across the whole strand, long mRNAs will have more cuts than short mRNAs and therefore resulting reads are more biased towards the 3′ end.

**Figure 3:**
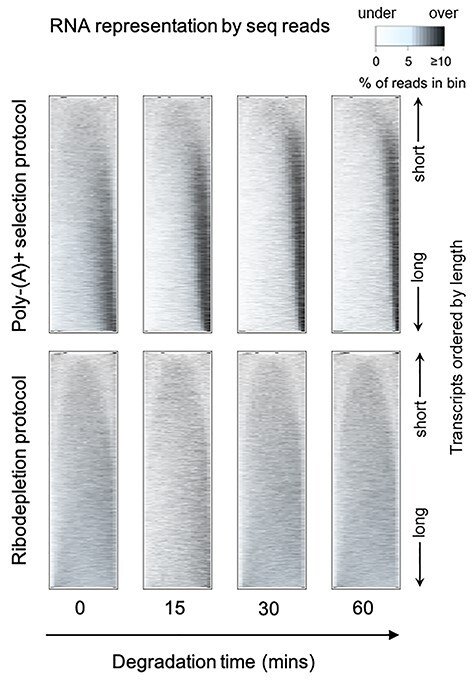
Biases in experimental degradation datasets of human brain tissue illustrated by heatmaps (data from NCBI BioProject. Accession number: PRJNA389171, brain number: Br1385). Samples were left at room temperature to degrade for different amounts of time and both poly(A) + and ribodepleted libraries were sequenced. Increasing degradation times typically results in a more pronounced coverage bias towards the 3′ end of the transcript for the poly(A) + but not the ribodepleted libraries.

To assess this bias, we used a collection of RNA-seq degradation datasets of human dorsolateral prefrontal cortex tissue [29] that had been prepared using either poly-(A) + selection or ribodepletion. Standard ribodepletion based protocols work by removing ribosomal RNA through sequence specific hybridization followed by bead separation or enzymatic degradation. Inspection of read distribution heatmaps (Figure 3) for these datasets show a more pronounced 3′ bias with increased degradation times, particularly with longer genes with pA+ selection, but not with ribodepletion. This also suggests that 5′ to 3′ exonucleases do not cause the bias on the poly-(A) + selected samples, because if they did, the bias would also be present in the ribodepleted samples.

Another major difference between RNA-seq protocols concerns the strategies for producing cDNA. The first step, RT, is initiated from primers that are designed to either bind random positions or that target the 3′ poly-A tail of mRNAs. ‘Random-priming’ was and is common in RT-PCR and is used in strand-specific RNA-seq systems such as the ScriptSeq or Ovation kits (Illumina and NuGEN, respectively), while the latter, ‘oligo-(dT) priming’ is very popular for scRNA-seq. This is because the primers will not target rRNA and therefore eliminate the need for purification of mRNA, thus potentially reducing losses of the limiting starting material.

Depending on the protocol, second-strand synthesis may once again start from a random position or from the terminus of the first-strand. The enzymes used in these reactions, reverse transcriptase and DNA polymerase, are both processive. This means that large numbers of nucleotides are incorporated before the enzyme drops off or the reaction stops otherwise (e.g. by reaching the end of the template strand). However, the exact stopping points cannot be predicted and are best described as probabilities for certain positions.

These positional dependencies between first- and second-strand priming cause global biases which have been noticed early [30]. Attempts to fully understand these are scarce, presumably owing to their complexity, but approaches to experimentally tackle them have been developed. One way to strongly reduce cDNA bias is to perform fragmentation on the original RNA instead of the cDNA. Since the resulting fragment length (~200 bp) is usually an order of magnitude shorter than the enzyme processivities [31], internal synthesis stops become negligible. The resulting coverage is more uniform on a global scale as simulated in the top row of heatmaps in Figure 2. However, RNA fragmentation is not practiced with scRNA-seq; it risks degradation of RNA and requires ligation of the first-strand primer directly to RNA, which is presumably of low efficiency since no scRNA-seq study has done it. There does not appear to be a reference for this, though, and an actual investigation of this might be prudent [32].

**Figure 4:**
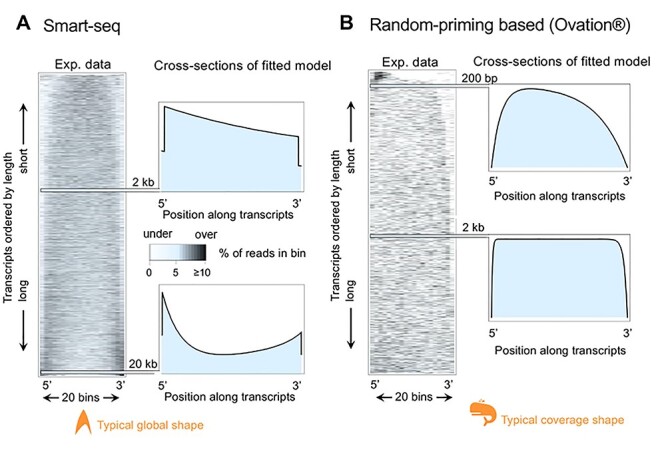
Biases in experimental datasets as illustrated by heatmaps and sections of fitted theoretical models. (**A**) Typical bias resulting from a Smart-seq based dataset (Encode project accession number ENCSR096STK). Heatmap (left) as in Figure 3 LiBiNorm software [36] was used to fit a bias model to the data whose predicted coverages are shown for two transcript lengths (right). (**B**) As A, for a typical random priming based (Ovation® system, NuGEN; GEO accession number GSE84724) dataset. Typical global pattern and coverage shapes are indicated in orange for Smart-seq and random priming in A and B, respectively.

The protocol-specific global bias is thus hard to experimentally avoid for scRNA-seq. However, it can be understood and thus corrected based on its shape. In fact, the shapes of the global bias are highly characteristic for the library preparation protocol that was used; datasets prepared with ‘SMART’-mechanism-based protocols (‘Smart-seq’ and its derivatives, see below) [33], which are prominently employed in scRNA-seq applications, resemble a Star Trek insignia on the heatmap; its bias shifts from central to bimodal with increasing mRNA length (Figure 4A). Subsequent improvements in this protocol resulted in Smart-seq2 [34], shows the same global bias. We envision the recently published Smart-seq3 [35] with further improvements in enzymes and buffers as well as the addition of a 5′ UMI will also show a similar bias shape as the mechanisms resulting in the bias remain (see below). Datasets based on random-primed first strands, as implemented by the Ovation kit (NuGEN), for instance, display a faint ‘ridge’ slightly off center that diminishes with increasing transcript length. Coverage profiles at different lengths might be described as having whale-like shapes (or perhaps the hat from ‘The Little Prince’), Figure 4B. A summary of the major types of biases and their effects is shown in Table 1.

**Table 1:** Different types of biases and their properties. The information was collated based on basic logics, heuristic considerations and literature examples where it was available. Note that some issues addressed in this review have not been systematically researched yet

Types of bias	Visibility in coverage	Local	Global	Non-linear length-scaling	Strength of bias	References
Fragmentation efficiency	Yes	Yes	Yes	Yes	Moderate	[23]
PCR	No	No	Yes	Potentially	Strong	[123]
Random priming	Potentially	Yes	No	No	Moderate	[23]
Sequence-specific	Yes	Yes	No	No	Small	[25] [24]
Processivity random priming	Yes	No	Yes	Yes	Moderate	[23] [30]
Processivity SMART	Yes	No	Yes	Yes	Strong	[30] [23, 36]
RNA degradation	Yes	No	Yes	Yes	Strong	[28], this review

The effect of these non-linear length-dependent global biases is to miscalculate expression levels when assuming a linear relationship between expression levels and transcript length (e.g. TPM, RPKM, FPKM). For example, using poly-(A) + selection on degraded RNA would result in the underestimation of long transcripts expression levels as these are missing more reads at the 5′ end than shorter transcripts. In a similar fashion, libraries based on Smart-seq also underestimate the expression of long transcripts [23] (see Section Global bias estimation). The size of this effect can be quite dramatic, for example Dyer *et al*. found if a FPKM/TPM is used to compare short (200 bp) and long (20 000 bp) transcripts on a dataset prepared with smart-seq2, there would be a ~9-fold error [36]. In some experiments, this bias could be less important than others. One example could be an experiment designed to discover differentially expressed genes between two conditions using poly-(A) + selected mRNA. A common pipeline would be to generate counts (the number of sequencing reads overlapping each gene) and pass this into a differential expression tool. Here, the global bias due to RNA degradation might affect both samples equally and not affect which genes are differentially expressed. However, if degradation is different between samples – the global bias will affect quantitation. Of course, for experiments measuring expression between genes in the same sample these global biases will have a large effect [23, 36].

One way to tackle bias is via spike-in controls [37]. Spike-ins are RNA molecules of known concentrations and lengths that are added to samples at early stages of library preparation. After sequencing, the reads aligning to these spike-ins can be then compared to their known concentrations to correct for differences in library preparation efficiency between samples (see section Technical noise in scRNA-seq for further information on the uses of spike-ins in (sc)RNA-seq).

In theory, the length-dependent global biases present in bulk and scRNA-seq could also be monitored and corrected by using spike ins. This could be achieved by comparing known concentrations against sequencing reads for different lengths of spike-ins and adjusting sequencing reads up/down depending on their lengths [23]. Unfortunately, these spike in probes are fairly short; for instance, ERCC probes, which dominate use in existing datasets [37], are roughly between 250 and 2000 nucleotides in length and so cannot be currently used for measuring global bias in long RNAs. Longer alternatives are now becoming available, though [38].

#### Analysis approaches to combat bias

Multiple computational methods have been developed to combat some of the biases in RNA sequencing experiments to more accurately quantify expression levels in coverage-based protocols. As mentioned before, the spiky peak landscape arising from local biases may not have a strong effect on quantitation, particularly in longer genes where this might average out – we do note that isoform quantitation will be affected by this, though [39, 40].

For a gene with only one isoform and in the absence of any sources of bias, coverage would be uniform across exons. Local and global biases mean this often is not the case. Roberts *et al*. (2011) address the local bias by redistributing reads within a transcript to make the coverage more uniform. Many tools either implement this strategy directly (Cufflinks [41], Salmon [40]) or take similar approaches [17, 42, 43]. A problem with this course of action is that it ignores the non-linear global length dependent bias and does not change expression estimates for single isoform genes. This results in the underestimation of long genes’ expression levels for SMART and poly-(A) + selected samples [23].

Some protocols use random hexamers to prime RT, which causes sequence specific bias [25]. This results in preferential sequencing of fragments starting with particular motifs and cannot be corrected by simply trimming the ends of reads as this will result in the bias shifting to sequences next to the random hexamer bias. The standard approaches to tackle this are algorithms that try to ‘learn’ bias patterns, that is, find sequences associated with lower or higher count density around the start of a read, and then adjust read counts up or down accordingly. Examples for this include variable length Markov models [44] (implemented in Cufflinks [41], kallisto [45] and Salmon [40]), Bayesian networks (seqbias [46]) and recurrent neural networks [47].

GC content can also contribute to under-representation of sequences, presumably due to incomplete PCR amplification, which can also be modeled and corrected for [39, 40].

#### Global bias estimation

There are few tools which attempt to understand and correct for global biases. Maxcounts [48] tries to avoid this bias altogether by taking the maximum number of overlapping reads that is found at a position along a transcript to measure its expression level. The downside of this approach is its rejection of the majority of sequencing reads, which could otherwise provide useful insights, and its potential vulnerability to local biases [36].

Wan *et al.* [49] model the non-linear global bias as an exponential decrease from the 5′ end, which appears to be an oversimplification, though, given the presence of 5′ bias, or clear bimodal biases on both the 5′ and 3′ ends of many datasets (Figure 4). The Flux simulator tool [50], can simulate enzymatic reactions in library preparation and *in silico* reproduces aspects of the global bias, but does not provide any bias correction and uses a model with some shortcomings [23].

Our group has developed the tool LiBiNorm [36] which follows from the work of Archer *et al*. [23] and fundamentally differs from the vast majority of existing software tools for bias correction. Here, the probabilistic aspects and logics of the enzymatic conversion steps of different protocols are taken into account, which allow the construction of mathematical models that predict certain coverage shapes. Fitting the predicted coverages to datasets yields parameter values for characteristics of the RNA-seq library preparation process, such as the processivity estimates. This information can be used to derive improved estimates for the relative expression levels of the original mRNAs. Importantly, this approach is based on inference of reaction mechanisms underlying the library preparation, which thus provides biochemical reasons for systematic under- or over-representation of transcripts by sequencing reads.

This is exemplified by Smart-seq and similar SMART-based protocols, where underrepresentation of long transcripts is expected based on the library preparation logics; SMART refers to the ‘Switching Mechanism At the 5’ terminus of the RNA Transcript,’ which introduces the second-strand primer at the end of the first-strand [33]. Due to the SMART mechanism and a PCR selection step [51], incomplete first strands, where RT fails to reach the mRNA’s 5′ end, are not targeted for PCR amplification. This occurs more frequently for long mRNAs, which get depleted in the process.

The protocol also leads to more even coverage, which serves to render the global bias less obvious.

However, imperfections in the protocol such as the spurious occurrence of the SMART mechanism inside of some transcripts (and not at 5′ ends only), usually yield non-uniform coverage of the observed shape (Figure 4A). This allows fitting models which produce estimates for enzyme processivities and similar parameters, in turn allowing for correction of the length bias and also providing a way to diagnose potential library preparation issues.

While local bias is too strong to permit precise bias estimation in many cases, we found LiBiNorm to perform well on Smart-seq2 datasets; these are prepared using additional measures to reduce local bias [34] and allow improved quantitation upon LiBiNorm processing and global bias correction [36].

A summary of bias correction tools is in Table 2.

**Table 2:** Selection of bias correction software tools. The list is intended to give an overview of the landscape and is not exhaustive and subject to limitations in the descriptions etc

Name	Protocol type	Bias type addressed	Comments	Reference
LiBiNorm	Coverage based, specifically Smatseq2	Global	Only current tool that addresses cDNA-related global bias	[36]
Wan *et al*.	Coverage	Global	Interprets global bias as RNA degradation only	[49]
Flux simulator	Coverage	Local and global	Captures some simplified features of global bias, but only for simulation, not correction; only model that at least in principle considers cDNA priming/synthesis as bias source	[50]
RNASeqBias R package	Coverage	Local and global	Assumes independence between expression level and gene/RNA length and thus corrects globally	[24]
Sailfish	Coverage	Local and global	Method is based on [24]	[124]
AIDE	Coverage	Local	Focus on isoforms	[125]
BCseq	Coverage, specifically scRNA-seq	Local and dropouts	Focus on scRNA-seq and addresses dropouts	[126]
Bento-seq	Coverage	Local	Focus on splicing	[127]
iReckon	Coverage	Local	Focus on isoforms	[128]
kallisto	Coverage	Local	Focus on sequence specific bias, uses a similar method to [44]	[100]
Maxcounts	Coverage	Local	Novel approach; appealing in its simplicity; limited in its power	[48]
Mix2	Coverage	Local	Focuses on positional biases using mixture models, closed source C++ implementation	[52]
CEM	Coverage	Local	Focus on isoforms and transcriptome assembly	[129]
Howard and Heber	Coverage	Local	Focuses on positional biases for isoform quantitation	[42]
Wu *et al*.	Coverage	Local	Focus on isoforms	[43]
Huang *et al*.	Coverage	Local	Focus on isoforms	[130]
Liu *et al*.	Coverage	Local	Focus on sequence specific bias	[131]
Alnasir and Shanahan	Coverage	Local	Focus on sequence specific bias	[132]
Zhang *et al*.	Coverage	Local	Employs deep learning for sequence specific bias correction	[47]
Jiang and Salzman	Coverage	Local	Focus on isoforms	[133]
Roberts *et al*.	Coverage	Local	Implemented in several software tools (CuffLinks, kallisto, etc.) in various iterations	[44]
NLDMseq	Coverage	Local	Focus on isoforms	[134]
PBSeq	Coverage	Local	Focus on positional and sequence specific biases	[135]
PennSeq	Coverage	Local	Focus on isoforms	[136]
PGseq	Coverage	Local	Considerers positional and sequence specific biases	[137]
PM-seq	Coverage	Local	Uses mixture models	[138]
RSEM	Coverage	Local	Concentrates on positional bias	[17]
Salmon/Alpine	Coverage	Local	Uses the method of [44] for positional and sequence specific bias, with additional GC bias correction	[39]
seqbias	Coverage	Local	Concentrate on sequence specific bias	[46]
Sequgio	Coverage	Local	Focus on isoforms	[139]
SparseIso	Coverage	Local	Focus on isoforms	[140]
WemIQ	Coverage	Local	Focus on isoforms	[141]
XAEM	Coverage	Local	Focus on isoforms	[142]
bayNorm	scRNA-seq	Dropouts	Bayesian approach and non-zero inflated binomial distribution	[111]
MAGIC	scRNA-seq	Dropouts	Dropout recovery by sharing information from neighborhood cells	[108]
Qju *et al.*	scRNA-seq	Dropouts	Cell classifier based on dropout co-occurrence	[112]
SAVER	scRNA-seq	Dropouts	Empirical Bayes approach for dropout imputation based on intergenic correlation	[83]
scImpute	scRNA-seq	Dropouts	Bayesian approach to rescue dropout gene using information from similar cells	[109]
ScVI	scRNA-seq	Dropouts	Neural network approach for scRNA-seq data processing	[87]
ZIFA	scRNA-seq	Dropouts	Dimension reduction accounts for dropouts	[85]
ZINB-WaVE	scRNA-seq	Dropouts	Imputation method based on zero-inflated model	[86]
Buttner *et al*.	scRNA-seq	Batch correction	Benchmark batch correction methods	[93]
Xi *et al.*	scRNA-seq	Doublet discrimination	Benchmark doublet discrimination methods	[96]
SoupX	scRNA-seq	Ambient gene expression	Use empty droplets to learn model	[97]
EmptyDrops	scRNA-seq	Empty droplets	Model ambient RNA pool to detect empty droplets	[100]

#### (Faux) ‘Gold standards’

Many bias correction methods use RT-PCR as a gold standard to measure the success of the correction and benchmark data to other tools. However, RT-PCR involves cDNA production as well and is therefore subject to the global bias, too. Employing a different gold standard might be advisable, such as datasets prepared with the ‘TruSeq’ protocol that fragments RNA (not cDNA), thus reducing the bias in RT (although we note here that RNA degradation can still cause 3′ bias in TruSeq samples – Figure 3), or data derived from RNA fluorescent in situ hybridization (FISH) or the Nanostring nCounter® system. In fact, some popular bias correction tools (Cufflinks [41], Mix^2^ [52]) are effective when RT-PCR is used as (faux) ‘gold standard’ but often perform worse than even simple linear TPM when benchmarking on RNA-fragmented data (e.g. TruSeq) [36].

#### Model-driven insights

Ideally, the model and tools employed will be able to correct biases and provide insight into how the latter occur. This will help to develop experiments to test these insights, enabling better understanding of the biology and make improvements into RNA sequencing protocols based on model predictions – a particularly pressing issue to resolve the zero-inflation controversy in scRNA-seq (see section Dropouts). Whilst most tools make little attempt to explain these technical issues, some do – which we will briefly touch upon.

For example, the *global* bias is interpreted as RNA degradation in Wan *et al*. [49]. This makes the prediction that increased degradation conditions will increase 3′ bias, and whilst this is true for the poly-(A) + selected samples, it is not for the ribodepleted samples (Figure 3). This suggests that degradation does not only occur from the 5′ end.

The approach taken by Archer *et al*. [23] showed that careful modeling of the sample preparation (in this case enzyme processivities) admits to tests of the model; modulating reaction conditions in one part of the protocol (altering reaction temperatures for first or second strand synthesis) changed the resulting global bias in line with predictions of the underlying model. Exploiting these insights allowed improving the experimental protocols, in this case by boosting enzyme processivity via decreased reaction temperatures. This in turn improves conversion of RNA into cDNA, along with a reduction of global bias, and was adapted by subsequent studies on full-length RNA sequencing protocols (e.g. RamDA Seq [53]).

### UMIs

**Figure 5:**
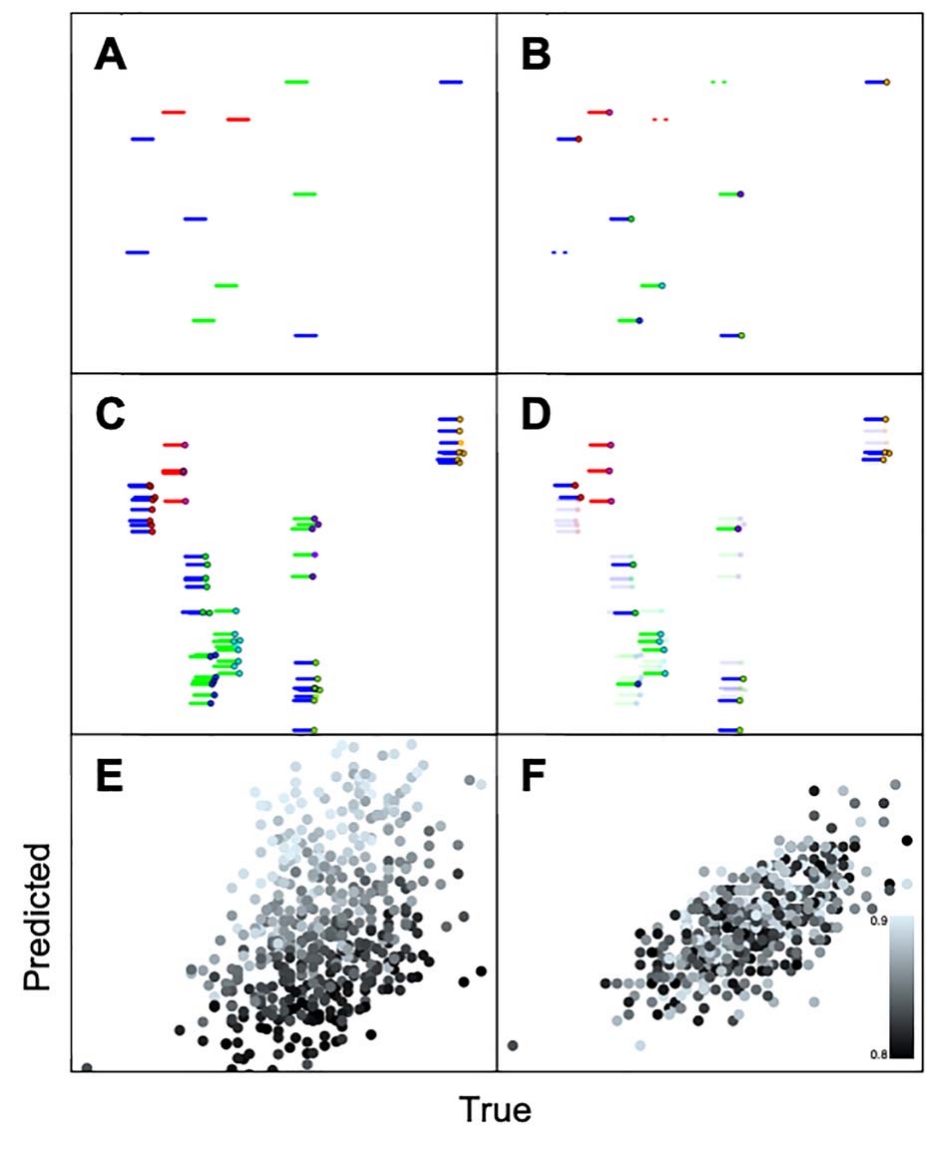
Schematic illustration of RNA library preparation and sequencing using UMIs produced using simulations, similar to [55]. (**A**) Initial number of RNA molecules in sample, colors indicate different genes. (**B**) Molecules get ‘captured’ by UMI tags (dots). Non-captured RNA molecules are lost (dashed lines). (**C**) Captured molecules are amplified by PCR. (**D**) Sequencing of cDNA library, sequencing depth determines how many copies are lost (transparent). (**E**) Reads to mRNA counts relationship is noisy due to stochastic effects during amplification and sequencing, as well as PCR efficiency variation between genes. Color brightness indicates PCR amplification efficiency, with darker colors indicating lower efficiency. (**F**) Sequenced UMIs are used to remove duplicate reads, improving the estimation of the initial RNA molecules.

UMIs are random oligonucleotide tags which are designed to label each individual mRNA molecule. When the length of the random oligos increases, there is an exponential increase in the number of possible distinct UMIs (4^λ^, where λ = UMI length), meaning that each RNA is very unlikely to be tagged with the same sequence. When sequenced, reads bearing the same UMI are counted once only, thus removing the potential bias of unequal PCR-amplification [54]. Ideally this allows inferring absolute molecule numbers [54], although in reality molecules are often under/over-estimated [55]. The process of using UMIs and their effect on the data can be illustrated using simple simulations of the library preparation process [55], as shown in Figure 5.

This notion has to be treated with some caution though, as rates of reaction are what determine biochemical processes, and these are defined by concentrations and not numbers of transcripts [56]. Thus, differences in transcript numbers between cells could be due to a cell size difference (an interesting phenotype in its own right) with no changes in rates of reactions. Ideally this would be accounted for, and whilst it is possible on the scale of a few genes with RNA FISH, through microscopy [57] or flow cytometry [58] and even transcriptome-wide with barcoding [59] – this is laborious and expensive. Recently, scRNA-sequencing combined with cell imaging measurements using microfluidic devices has been demonstrated [60, 61].

To prevent ‘over-counting,’ multi-labeling of single transcripts with different UMIs must be avoided. This makes the poly-A tail a preferred target for labeling an individual mRNA uniquely using oligo-(dT) – UMI concatenates, thus restricting detection to poly-(A) + transcripts. There is also a trade-off with sensitivity; UMI usage restricts quantification of a transcript to the single fragment (usually from the RNA’s 3′ end) bearing the UMI, whereas other fragments are lost. Therefore, for studying any processes away from the 3′-end, such as alternative splicing, UMI-based methods are not useful. This problem may be solved in the future with greatly increasing sequencing lengths, ideally resulting in full length UMI labeled transcripts being sequenced. For now, coverage based methods (or combining coverage based methods with UMIs [35]) still have to be used to answer these types of questions.

Restricting this quantification to the single fragment at the 3′ end means that the global length dependent biases of degradation and processivity are irrelevant. However, whilst we have said that local biases might not have a strong effect on quantitation for coverage-based protocols, the opposite may be true for UMI based ones. This is because restricting quantitation to only a single fragment means that local peaks or valleys in that fragment will not be averaged with reads from the rest of the transcript (unlike coverage-based protocols). Worse still, it will be an invisible type of bias.

## Single cell

Sequencing at a single cell level has recently gained huge traction in a variety of fields (see [62] for a review). The major advantage of scRNA-seq over bulk RNA sequencing is that the identity of the individual cells in a population is preserved. This allows for a heterogeneous population such as those found in biomedical samples to be dissected into its constituent sub-populations after sequencing, which can be used to detect diseases at early stages [63–65], or track the progression of differentiation and development [66–68]. Similarly, differences in the expression levels between cells of homogeneous populations can be measured, which can be useful for interpreting the underlying stochastic mechanisms of gene expression. Therefore, mRNA distributions that can be obtained with scRNA-seq are a much richer source of information than the average RNA expression conferred by bulk RNA sequencing.

UMIs are particularly useful in scRNA-seq, where PCR amplification efficiency varies between single cells as well as between genes. For this reason, the next section is focusing on single cell sequencing methods using UMIs. However, it is important to note that the same technical effects also apply to coverage based single cell studies.

### Technical noise in scRNA-seq

scRNA-seq suffers from several additional sources of technical noise, which contribute to the observed variation between single cells [69, 70]. The first relates to the sampling or ‘counting’ error associated with the number of RNA molecules captured by the library preparation process. This results in an intrinsic source of technical noise which poses a limit to the precision of scRNA-seq. While sampling error is present in bulk RNA-seq too, it is negligible in practice due to the much higher amount of input material. In contrast, sampling error gains significance in scRNA-seq due to the low number of RNA molecules per cell. Modeling techniques can be used to account for this source of technical noise [70, 71].

In addition to sampling error, scRNA-seq suffers from variation in the library preparation efficiency between cells, resulting in a variable fraction of RNA molecules per cell being converted to cDNA [46]. This can be due to subtle differences in the concentration of primers and library preparation enzymes between cells, as well as variation in cell lysis efficiency [47]. As a consequence, a technical source of variation is introduced into the total size of single cell cDNA libraries. This is another type of error which is well known from bulk RNA-seq, where it is accounted for by expressing RNA counts in terms of reads per million (RPM) [72]. In scRNA-seq, the total RNA per cell is often assumed to be constant, and the libraries are scaled based on a group of genes which are assumed to be stably expressed [73, 74].

However, relying on such normalization methods ignores the likely variation in transcriptome size between biological groups, or single cells in the case of scRNA-seq, which is a natural source of library size variation. As highlighted by others [72, 75], doing so can lead to vastly different interpretations of the data. Ref. [76] showed that by accounting for changes in transcriptome size, more than 6000 genes were found to be induced during yeast aging, as opposed to only the few hundred identified previously [77]. The implications are even greater for scRNA-seq, where often the aim is to compare the expression between cells in a heterogenous population, consisting of cells of different type, volume and cell-cycle stage, all of which are expected to affect the natural size of the transcriptome [78].

Correcting for technical variation in library size is therefore a crucial step in the pre-processing of scRNA-seq data. Similar to bulk RNAseq, this can be achieved by using an internal [79] (e.g. housekeeping genes) or external (RNA spike-ins) reference point [69], with respect to which the individual libraries can be scaled. In the former case, a certain group of genes which is assumed to be non-differentially expressed between cells is defined, and any variation in the number of counts for these genes is ascribed to technical sources, allowing the libraries to be scaled accordingly. The validity of this assumption however is not always easy to ascertain, especially in single cells, where gene expression stochasticity can lead to a variable degree of expression even for stably expressed genes [80, 81]. This method is preferred in droplet-based methods, where the application of spike-ins is unfavorable (see below and [81]).

Using RNA spike-ins, the library sizes can be normalized between cells without requiring any assumptions about their gene-expression profile. The assumption underlying the use of spike-ins is that the same amount is added to each cell, and that the variation in capture efficiency between cells is similar for endogenous and spike-in RNA. While the use of spike-ins has been criticized for use in bulk RNA-seq [82], systematic analysis in plate-based scRNA-seq has shown that they are a reliable method for normalization [81].

Using RNA spike-ins for normalization also has certain limitations. For example, as spike-ins can only be added once the cells have been lysed, they do not reflect the error arising from variation in lysis efficiency between cells [71]. Furthermore, a pilot experiment is advisable to determine the optimal amount of spike-in RNA to be added (5–10% of library size) [81]. Criticism regarding the commonly used ERCC spike-ins [37], whose gene and polyA tail lengths are shorter than many endogenous transcripts, have also been raised [83]. These however are mostly relevant to absolute quantification of mRNAs rather than library normalization between cells. In either case, spike-ins remain the only way currently to normalize between single cell libraries without making strong assumptions about gene expression variation between cells, and have therefore been strongly recommended for this purpose [84].

Perhaps the biggest limitation of spike-ins is that they cannot easily be used with current droplet-based scRNAseq, thus limiting their use to plate-based scRNA-seq [81]. This is in part because the highly diluted cell suspension required to minimize the number of doublet encapsulations results in a high fraction of empty droplets. In the absence of spike-ins, the empty droplets do not contribute to the sequencing cost. When spike-ins are used however, spike-in cDNA is produced for every single empty droplet, which can double the cost of sequencing [85]. Recently, a drop-seq device which enables ordering of the cells into a line prior to encapsulation has been shown to achieve much higher working concentrations of cell suspensions and thus fewer empty droplets [86]. Improvements in this area are likely to make the use of spike-ins in droplet-based sequencing more cost-effective.

Due to the popularity of droplet-based scRNA-seq methods (see [87] for a review), driven in part by the lower cost compared to plate-based scRNA-seq, several spike-in free normalization methods have been proposed which account for the natural variation in transcriptome size. By modeling the RNA molecule capturing process by UMIs and by randomly assigning plausible capture efficiencies to each cell, Ye *et al*. [88] produced estimations of the molecule counts per cell without spike-ins, which were comparable to results from spike-in based normalization. However, a requirement of this method is that genes are assumed to have a zero-inflated (see below) negative binomial distribution, which may not hold for all genes, conditions and systems. Instead, Wang *et al*. [78] use a more flexible prior for the gene-expression distribution, allowing for the shape of the biological distribution to be inferred while accounting for changes in the transcriptome size. Systematic comparisons between these and other methods are required to establish when each is most suited for.

Other sources of technical noise affecting scRNA-seq include batch effects, the presence of doublets and multiplets, ambient gene expression and gene dropouts, the latter of which is discussed in more depth in the next section. Accounting for these effects is part of the standard pre-processing and quality control of most scRNA-seq experiments, an overview of which can be found in [89].

Batch effects, also known from bulk RNA-seq, occur when cells from different biological groups are processed separately. In such cases, technical variation during each step of the process (cell culture, capture and sequencing) introduces biases which, if not accounted for, can confound data analysis [90]. The best way to deal with batch effects is to design the experiment in a way that avoids them all together. For example, batch effects can be avoided when using plate-based scRNA-seq by ensuring that cells from each biological group are equally represented on each plate [91], something that can be easily achieved using fluorescence activated cell-sorting.

This is not possible for all scRNA-seq protocols, however. Specifically in droplet-based sequencing, the standard balanced experimental design cannot be easily achieved as cells need to be encapsulated and sequenced separately for each sample, in order to retain each sample’s identity [90]. Of note are recent developments in cell-tagging methods (cell ‘hashing’), which allow cells from different samples to be pooled together prior to encapsulation and sequencing [92]. The reads from cells belonging to different groups can be subsequently demultiplexed, thus avoiding batch-effects altogether. In cases where this is not possible, correction needs to be performed at the analysis stage (see [93] for a comparison of existing methods, also [94]).

Doublets result when two cells are co-encapsulated in the same droplet or land in the same well of a multi-well plate in the case of plate-based scRNAseq. The result is that reads from these cells cannot be de-multiplexed, leading to artificial transcriptomes in the data. While these can often be identified by their unusually high number of associated transcripts, inherent variation in transcriptome size often found in cell populations means arbitrarily setting a threshold also introduces a bias [95]. Recent computational models developed to account for the presence of doublets in an unbiased way are compared in [96].

Ambient gene expression refers to extracellular RNA which becomes encapsulated into the same droplet as a cell or accompanies a cell in the same well, leading to contamination of a cell’s resulting transcriptome. The presence of extracellular RNA in the sample results from RNA leaking from damaged cells during the sample preparation process, and unless it is accounted for leads to biases in the interpretation of the data. While it is difficult to completely remove non-endogenous RNA from the sample prior to library preparation [9], novel methods exist which can filter these RNA molecules at the analysis stage.

Methods such as SoupX [97] use the existence of empty droplets (or wells) to calibrate a cell-free RNA model, which can be used to correct the data. In the case of plate-based scRNA-seq, this can easily be achieved by sequencing several wells into which only cell suspension buffer has been dispensed. For droplet-based sequencing on the other hand, distinguishing empty droplets is not a trivial task. While a common approach is to set a threshold on the minimum number of RNA counts [9, 98], this introduces another bias.

Specifically, certain types of cells (usually smaller cells) will also have fewer counts than the average cell and can thus be mistakenly excluded from the analysis. The opposite problem also exists – empty droplets containing cell-free RNA can be mistaken for a distinct cell type. This has motivated the development of methods which model the profile of empty cells to efficiently exclude them from analysis [99, 100] without removing genuine cells with low RNA counts. Combining such an approach with existing models for cell-free RNA such as SoupX could be a powerful way to tackle both filtering of empty droplets and cell-free RNA from the data.

### Dropouts

Because of the finite size of the single cell starting material, often genes with moderate or low expression level will not be detected, which leads to the over representation of zero counts of gene expression in the final scRNA-seq datasets. This ‘dropout’ phenomenon has been an actively discussed topic since the first emergence of the scRNA-seq itself [70, 101, 102] and is commonly ascribed to technical reasons (e.g. capture efficiency, sampling noise or PCR bias) and deemed an obstacle for quantitative analysis [103].

(While this section has a focus on UMI methods, low capture efficiency can affect coverage-based single cell RNA sequencing datasets too. For example; in a particular cell, a gene with two isoforms, both expressed at the same level, can wrongly appear as though only one isoform is being expressed in scRNA-seq experiments with low capture efficiency. This is especially important when the expression levels are low [104].)

It is thus common to process scRNA-seq data using zero-inflated distribution models for deconvoluting meaningful biological variance from high counts of zeros due to technical noise. Therefore, many ‘imputation’ algorithms have been proposed to ‘rescue’ scRNA-seq data from the inflated zero counts for downstream applications [78, 101]. For instance, dimension reduction based on zero-inflated distributions was used on scRNA-seq datasets to extract informative variables for further analysis (e.g. ZIFA [105], ZINB-WaVE [106] or ScVI [107]). Other imputation methods such as MAGIC [108], SAVER [102] or scImpute [109] ‘fill in’ the undetected RNA counts by exploiting gene–gene expression relationships and information from neighboring cells sharing similar expression profiles. Although these approaches are potentially powerful tools to address the dropouts problem, their proclivity to introduce artifacts [109] and/or erase existing differences by over-smoothing the data is known [102].

Recently, it was argued by Svensson [110] that at least for a range of droplet-based UMI scRNA sequencing methods (e.g. 10x Chromium [9], Drop-seq [10] or InDrop [11]), complex zero-inflated model based analysis might not be necessary. The dropout events (zero counts) could be explained well enough by rather simple Poissonian distributions (gamma-Poissonian mixture or negative binomial distribution) and correspond to biologically meaningful information rather than technical noise, according to the author. Based on this simpler binomial distribution and taking a Bayesian approach, Tang *et al*. [111] developed bayNorm – an integrated package for processing scRNA-sequencing data and showed accurate reconstruction of experimental data by their simulation.

However, this might hold true only for the latest generation of UMI low-volume emulsion techniques as opposed to earlier methods, owing to improvements in capture efficiencies. All the same, high-dropout rates are still observed in modern methodologies and compatibility with Poissonian models does not prove genuinely biological origins. Moreover, the evidence collected from pure RNA control solutions might not convincingly explain sequencing data derived from cellular RNA samples, which exist in more complex environments.

The assumption that at least some of the zero counts reflect biological variance is supported by recent work from Qiu [112], who presented a cell type classifier based on dropout co-occurrence patterns alone. Furthermore, the author demonstrated that such a classifier is as powerful as classification algorithms based on high count mRNA molecules. This further supports the argument that dropout events contain meaningful biological information, rather than being purely an artifact.

### In conjugation with other techniques

A further issue with scRNA-seq datasets is illustrated well by the differing conclusions obtained from these compared to alternatives, such as single molecule RNA FISH (smFISH) [57] or live cell imaging [80]; whereas scRNA-seq data suggest that the vast majority of genes feature relatively little variability, consistent with a Poisson distribution of transcript numbers [11], imaging data usually shows variability higher than a Poisson’s (e.g. [58, 80, 113]).

One field of note here is spatial transcriptomics, where researchers combine measuring RNAs with their positional contexts. Here, imaging data such as smFISH appear well suited to the task, e.g. [114–116], however, as mentioned previously, it is difficult to measure more than a few genes simultaneously with this method. To combat this challenge, some methods use known positional information from ‘marker genes’ to calibrate results from scRNA-seq and define the spatial location of each cell [117, 118]. A problem with this calibration approach is that it requires prior knowledge of marker genes and their locations and can be biased by the choice of these genes. Technologies such as slide-seq [119] and Visium [120] allow the spatial transcriptome to be measured without using ‘marker genes’ by adding spatially barcoded RNA capture probes to a slide, upon which fresh-frozen tissue samples are placed. This results in cDNA containing the spatial barcode, which can then be used to assign the spatial location of the original mRNAs.

## Concluding remarks

(sc)RNA-seq is a rapidly maturing technology, with technical improvements continuing to increase the output in terms of numbers of samples/cells sequenced at an exponential rate [121]; it is now commonplace, with many easily accessible (commercial) implementations and bioinformatic tools to support data processing and analysis.

While for many biological questions, high sensitivity, precision or absolute quantification might not be necessary, biases are still present and underappreciated, which can skew the estimation of transcript abundances and influence the conclusions that are made.

Furthermore, for more complex aims such as analyses of non-genetic heterogeneity [6], gene regulatory network inference, or even quantitative descriptions of a whole cell [122], the best possible measurement of expression levels in all respects is required. Even in situations where relative expression levels only are of interest, fold changes can be meaningless if they concern very low expression levels, requiring estimation of the latter.

By highlighting them here, we hope researchers will consider these issues when designing experiments and continue to develop methods for dealing with them. These methods can be experimental, such as using spike-ins, employing UMIs to combat the PCR amplification bias and fragmenting RNA to combat RT bias, or computational, such as improving expression level estimation by modeling the sample preparation process.

**Key points**

- RNA-seq is subject to trade-offs between sensitivity and single transcript labeling
- Coverage bias can be local or global, visible or invisible
- Global bias can cause systematic and length-dependent over- or underestimation of transcripts, with protocol dependent patterns
- scRNA-seq in particular is affected by technical noise and dropouts
- These biases can be explained, understood, and partially corrected by novel analysis approaches

## Data Availability

The data underlying this article are available in Gene Expression Omnibus and NCBI BioProject at https://www.ncbi.nlm.nih.gov/geo/ and https://www.ncbi.nlm.nih.gov/bioproject/, and can be accessed with GSM710183, GSM710184 and PRJNA389171, brain number: Br1385.

## Notes

**Matt Jones** is a PhD student in Daniel Hebenstreit’s Research Group.

**Philip Davies** is a postdoctoral researcher in Daniel Hebenstreit’s Research Group.

**Juntai Liu** is a postdoctoral research fellow from a joint team at the Physics Department, University of Warwick.

**Daniel Hebenstreit** is reader at the School of Life Sciences at the University of Warwick. The group’s research interests are transcription, stochasticity, bioinformatics, genomics and next generation sequencing.
